# Oxidative Stress and Cancer Heterogeneity Orchestrate NRF2 Roles Relevant for Therapy Response

**DOI:** 10.3390/molecules27051468

**Published:** 2022-02-22

**Authors:** Koraljka Gall Trošelj, Marko Tomljanović, Morana Jaganjac, Tanja Matijević Glavan, Ana Čipak Gašparović, Lidija Milković, Suzana Borović Šunjić, Brigitta Buttari, Elisabetta Profumo, Sarmistha Saha, Luciano Saso, Neven Žarković

**Affiliations:** 1 Laboratory for Epigenomics, Division of Molecular Medicine, Rudjer Boskovic Institute, 10000 Zagreb, Croatia; marko.tomljanovic@irb.hr; 2 Laboratory for Oxidative Stress (LabOS), Division of Molecular Medicine, Rudjer Boskovic Institute, 10000 Zagreb, Croatia; morana.jaganjac@irb.hr (M.J.); acipak@irb.hr (A.Č.G.); lidija.milkovic@irb.hr (L.M.); borovic@irb.hr (S.B.Š.); zarkovic@irb.hr (N.Ž.); 3 Laboratory for Personalized Medicine, Division of Molecular Medicine, Rudjer Boskovic Institute, 10000 Zagreb, Croatia; tanja.matijevic@irb.hr; 4 Department of Cardiovascular, Endocrine-Metabolic Diseases, and Aging, Italian National Institute of Health, 00161 Rome, Italy; brigitta.buttari@iss.it (B.B.); elisabetta.profumo@iss.it (E.P.); sarmistha_pharmacol@yahoo.com (S.S.); 5 Department of Physiology and Pharmacology “Vittorio Erspamer”, Sapienza University of Rome, 00161 Rome, Italy; luciano.saso@uniroma1.it

**Keywords:** 4-hydroxynonenal, therapy resistance, cancer stem cells, tumor associated macrophages (TAMs), tumor associated neutrophils (TANs), polarization, *NFE2L2* promoter, KEAP-1, micro RNA

## Abstract

Oxidative stress and its end-products, such as 4-hydroxynonenal (HNE), initiate activation of the Nuclear Factor Erythroid 2-Related Factor 2 (NRF2)/Kelch Like ECH Associated Protein 1 (KEAP1) signaling pathway that plays a crucial role in the maintenance of cellular redox homeostasis. However, an involvement of 4-HNE and NRF2 in processes associated with the initiation of cancer, its progression, and response to therapy includes numerous, highly complex events. They occur through interactions between cancer and stromal cells. These events are dependent on many cell-type specific features. They start with the extent of NRF2 binding to its cytoplasmic repressor, KEAP1, and extend to the permissiveness of chromatin for transcription of Antioxidant Response Element (ARE)-containing genes that are NRF2 targets. This review will explore epigenetic molecular mechanisms of NRF2 transcription through the specific molecular anatomy of its promoter. It will explain the role of NRF2 in cancer stem cells, with respect to cancer therapy resistance. Additionally, it also discusses NRF2 involvement at the cross-roads of communication between tumor associated inflammatory and stromal cells, which is also an important factor involved in the response to therapy.

## 1. Introduction

It has been proposed that excessive production of reactive oxygen species (ROS) and numerous cellular redox adaptation responses are involved in cancer initiation, progression, and drug resistance [1,2,3]. Nevertheless, persistent oxidative stress renders tumor cells increasingly vulnerable to additional stressors and reverses resistance to treatment. Accordingly, redox perturbation could be instrumental in the selective elimination of cancer cells.

ROS (Reactive Oxygen Species) are oxygen-containing molecules formed by reduction/oxidation reactions (redox reactions) or electronic excitation. Key ROS molecules include hydroxyl and superoxide free radicals and nonradical molecules, such as hydrogen peroxide. When ROS production increases or their scavenging by antioxidants decreases, cells undergo a process of oxidative stress. Several growth factors and cytokines regulate ROS production in the mitochondria. This regulation is mainly via the electron transport chain, where oxygen is reduced to form superoxide anion [4], peroxisomes (through the β-oxidation of fatty acids) [5], and endoplasmic reticulum (through the oxidation of proteins) [6]. Exposure to exogenous agents, including radiation, heavy metals (especially transition metals such as iron, or metal complexes), atmospheric pollutants, and various chemicals (including xenobiotics and especially chemotherapeutic agents), leads to increased production of ROS [7,8].

Although potentially very harmful, even cytotoxic, ROS are crucial for cellular life. Namely, if present in moderate concentrations, ROS act as second messengers in the transduction of extracellular signals and in the control of gene expression related to cellular proliferation, differentiation, and survival [9]. At higher levels, ROS are also produced by cells as defense agents against pathogens [10,11,12]. Excessively high cellular levels of ROS can cause damage to proteins, nucleic acids, lipids, membranes, and organelles, which may lead to the activation of such cell death processes as apoptosis [13].

Several lines of evidence prove that ROS can cause DNA damage and contribute to occurrence of oncogenic mutations. Cancer cells, through their aberrant energy metabolism, commonly produce higher levels of ROS than normal cells. An increased level of ROS is associated with the activation of oncogenes, the inactivation of tumor suppressor genes, and mitochondrial malfunction [14]. Genotoxic stress has recently been shown to be a trigger of an inflammatory signaling cascade which results in the release of pro-inflammatory factors and an increase in the amount of infiltrating immune cells. These events additionally contribute to ROS production and lead to the occurrence of a vicious circle of carcinogenic oxidative stress [15].

Under such circumstances, ROS serve cancer as pro-growth signaling molecules, also triggering a self-catalyzed chain reaction process of lipid peroxidation of polyunsaturated fatty acids (PUFAs), in particular. The strongly induced peroxidation of PUFAs generates reactive aldehydes, molecules which are much more stable than ROS themselves. Therefore, they are considered to be “second messengers” of ROS [16]. Among such reactive aldehydes, 4-hydroxynonenal (HNE), the end-product of n6-polyunsaturated fatty acid peroxidation [16] is considered to be one of the most bioactive aldehydes. HNE has the ability to modify various cellular signaling pathways and processes [17]. It exerts its activity by binding to the cysteine, histidine, arginine, and lysine moieties of proteins changing their activity [18]. Additionally, HNE can bind to DNA, thereby causing mutations [16] but also to lipids (reviewed in [19,20]). The effects of HNE are concentration- and cell type-dependent [21,22].

**Figure 1:**
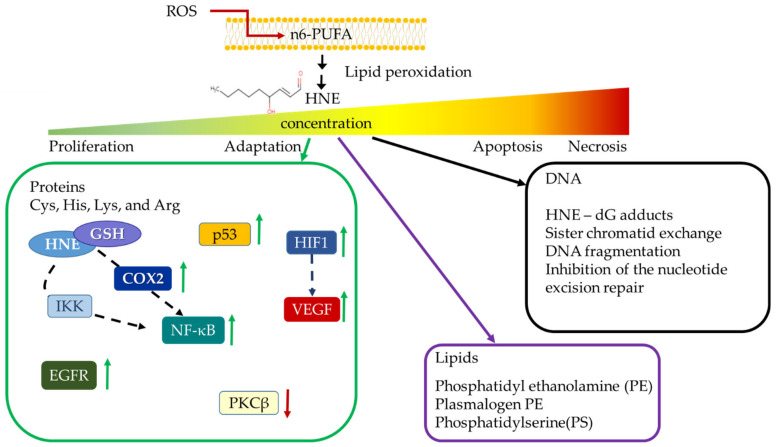
Interactions of HNE with cellular proteins, lipids and DNA. HNE is an end-product of n6-polyunsaturated fatty acids (n6-PUFA) peroxidation which acts in a concentration dependent manner. Low concentrations: interaction with DNA (black arrow) results in forming exocyclic guanine adducts [24]. High concentrations: occurrence of sister chromatid exchange, DNA fragmentation [19], and inhibition of nucleotide excision repair [25]. HNE also binds to membrane lipids (purple arrow) [20]. Interactions with proteins are much more complex, as HNE directly or indirectly causes an increase (green arrows) or decrease (red arrow) in the activity or expression of Nuclear Factor Kappa-Light-Chain-Enhancer of Activated B Cells (NF-κB), Cyclooxygenase 2 (COX2), Hypoxia-Inducible Factor (HIF1) Vascular Endothelial Growth Factor (VEGF), TP53 and Epidermal Growth Factor Receptor (EGFR) [17,26]. Interactions with NRF2 will be reviewed separately.

The type of response may be dependent on the metabolism of HNE. It is primarily detoxified through conjugation with glutathione (GSH). Thereafter, this complex is exported through RBP1 (RalA-Binding Protein 1) [23]. In low concentrations, HNE binds to proteins involved in signaling pathways or modulates proteins involved in the previously mentioned biological processes (summarized in Figure 1).

For maintaining redox homeostasis and limiting cellular damage, eukaryotic cells have developed mechanisms for the tight regulation of ROS levels. They are based on a complex scavenging system containing superoxide dismutases (SODs), catalases, thioredoxins, peroxiredoxins, and glutathione peroxidases [27,28,29,30]. Non-enzymatic antioxidants, such as glutathione (GSH), vitamin C (ascorbate), vitamin E (tocopherols), and polyphenols also act directly on ROS and other pro-oxidative agents [31].

However, some clinical trials and experimental models suggest that dietary supplementation with antioxidants, especially carotenoids and vitamin E, could increase cancer incidence and cancer-related deaths in humans [32,33,34,35,36]. On the one hand, this may be due to the abuse of these antioxidants, followed by an increase of their concentrations above the physiological level, and their conversion into metabolites which interfere with cellular metabolism [37,38]. On the other hand, cancer cells, in parallel with an increase of ROS, also increase their unique antioxidative capacities. In this way, cancer cells optimize ROS-driven proliferation and avoid ROS thresholds that would otherwise trigger cellular death [39,40]. 

In response to an excessive ROS production, cancer cells develop several transcriptional programs which rely on transcription factors/their binding partners that contain redox-responsive cysteines. These programs include members of the Forkhead Box Protein O3 (FOXO) family, Hypoxia Inducible Factors (HIFs), Kelch-Like ECH-Associated Protein 1 (KEAP1) with NRF2 and the Tumori protein P53 (TP53) tumor suppressor-related transcriptional program [41,42,43,44].

In healthy tissues, a transient activation of NRF2 has been long recognized as a cellular defense mechanism which is critical for preventing cancer initiation by carcinogens. The activation of the NRF2/KEAP1 signaling pathway allows cells to adapt and to survive under conditions of stress by regulating several diverse transcriptional networks. Their activation results in the synthesis of cytoprotective proteins, including antioxidant, anti-inflammatory, and detoxifying enzymes, as well as proteins that assist in the repair or removal of damaged macromolecules [45,46,47,48]. More than 1000 genes possessing an Antioxidant Response Element (ARE) in their promoters can be activated by NRF2 [49,50]. NRF2 signaling regulates cellular response to inflammation via suppressing pro-inflammatory cytokines and through controlling fundamental cellular processes such as apoptosis, autophagy, angiogenesis, proliferation, and cell migration [51].

Cancer cells frequently hijack the protective capability of NRF2 to sustain their redox balance and meet their proliferation-related metabolic requirements. Indeed, several types of cancer exhibit hyperactivation of NRF2, conferring not only a highly proliferative phenotype [52] but also bringing the onset of metastases [53]. In addition, the NRF2 increased activity confers on cancer cells resistance to commonly used chemotherapeutic agents and radiotherapy [54,55].

Among cancer cells, tumor cells with stem cell-like properties appear to have distinct redox profiles. These are intimately linked to tumor initiation, its cellular heterogeneity, maintenance, recurrence, and metastasis. Cancer cells are highly dependent on the tumor microenvironment (TME), that is composed of several kinds of stromal cells. These include cancer associated fibroblasts (CAFs), endothelial, adipose, and immune cells. A biunivocal relationship is established between cancer cells and TME. Cancer cells secrete several factors that induce TME to secrete other soluble molecules. In turn, these modify metabolism and redox state of cancer cells [56].

Since oxidative stress is considered an important metabolic stress that could limit as well as enhance the survival of cancer cells and impact the host’s response to cancer [57,58,59], NRF2 inhibition is considered an attractive treatment option to counteract the survival and proliferative advantage of cancer cells and reverse resistance to anti-cancer therapies [60]. In this review, we focus on molecular aspects of NRF2 functioning, with respect to its structure and modes of activation, associated with several aspects of oxidative stress and therapy response of cancer.

## 2. The NRF2 Structure and Regulation

According to the Human Genome Organisation (HUGO) Gene Nomenclature Committee (HGNC) and unless stated otherwise, italicized abbreviations for genes written in uppercase letters relate to human genes, while abbreviations written in lowercase letters relate to mouse and rat genes [61].

NRF2, encoded by the *NFE2L2* gene, is a member of the Cap‘n’collar (CNC) transcription factor family. It consists of 605 amino acids organized into seven highly conserved functional domains known as Neh1-Neh7 (NRF2-ECH Homology 1–7) [51]. The Neh1 domain has a cap ‘n’ collar basic-region leucine zipper (bZIP) domain, which regulates binding to DNA [62], and a nuclear localization signal (NLS) that is responsible for the translocation of NRF2 from the cytoplasm to the nucleus [63]. The Neh2 domain, an N-terminal regulatory domain, contains seven lysine residues and two motifs (DLG (low affinity) and ETGE (high affinity)) involved in the interaction with KEAP1, which is a negative regulator of NRF2 activity. KEAP1, influences both, the stability and ubiquitination of NRF2. Neh3 is responsible for the ARE activation. Both Neh4 and Neh5 are involved in the binding with different “cAMP (cyclic Adenosine MonoPhosphate) response element-binding” (CREB) proteins and are able to activate transcription [64,65]. The Neh6 domain, containing serine-rich residues, is a negative regulatory domain which binds to a β-transducin repeat-containing protein (β-TrCP), thus determining NRF2 ubiquitination [66]. 

The Neh7 domain inhibits the NRF2-ARE signaling pathway by promoting binding of NRF2 to the Retinoic X Receptor α (RXRα) and disrupting binding between CBP (CREB-binding protein) and the Neh4 and Neh5 domains [67].

KEAP1 is the negative regulator of NRF2 activation consisting of 624 amino acids. It is a cysteine-rich protein, containing 27 cysteine residues [68]. KEAP1 is divided into five domains: (1) an N-terminal region (NTR); (2) a Tramtrack and Bric-à-Brac (BTB) domain; (3) a central intervening region (IVR) with a nuclear export signal (NES) mediating the cytoplasmic localization of KEAP1 [69]; (4) six Kelch repeats, and (5) a C-terminal domain (CTR). Under unstressed conditions, the expression of ARE-responsive genes is kept at the basal level due to the retention of NRF2 in the cytoplasm by KEAP1/Cullin-3/E3 Ubiquitin-Protein Ligase RBX complex and subsequent proteasomal degradation [70]. Recently, p97, an Adenosine TriPhosphate (ATP)-dependent segregase, was identified as a canonical negative regulator of NRF2, needed for its efficient proteasomal degradation [71]. The high rate of NRF2 ubiquitination and degradation in non-stressed cells are largely cell-type dependent due to varying concentrations of the KEAP1 protein [72]. 

When exposed to stress-inducing endogenous or exogenous factors, cells activate the NRF2/KEAP1/ARE pathway [60,73]. Three cysteine residues, Cys151, Cys273, and Cys288, are important for KEAP1 functioning. Their modifications by electrophilic inducer activate the pathway. Although the mechanism is not completely elucidated, two models are proposed. The “hinge and latch” model proposes binding of the NRF2/KEAP1 by association of the low affinity DLG motifs of the Neh2 with one KEAP1-DC (double glycine repeat or Kelch, plus C-terminal) domain (latch) and the high-affinity ETGE motif with the other DC domain (hinge) [74]. According to this model, abolished NRF2 ubiquitination is a consequence of disrupted latch (DLG-KEAP1-DC), due to thiol modification [75,76]. Horie et al. [77] examined whether two KEAP1-NRF2 protein-protein interaction (PPI) inhibitors (PRL295 and NG262) and electrophilic NRF2-activating compounds (1-[2-cyano-3,12-dioxooleana-1,9(11)-dien-28-oyl] imidazole (CDDO-Im), sulforaphane (SFN), and 15-deoxy-∆-prostaglandin J_2_ (15d-PGJ_2_) utilize the Hinge-Latch mechanism. They also examined the p62/Sequestosome-1 in this mode of action. They found that KEAP1-NRF2 PPI inhibitors and phosphorylated p62/Sequestosome-1 peptide preferentially disrupt the KEAP1–DLGex binding, while CDDO-Im, SFN, and 15d-PGJ_2_ do not disrupt the KEAP1–DLGex nor the KEAP1-ETGE interactions These results suggest the existence of other mechanisms of NRF2 activation, in addition to the Hinge-Latch. SFN and CDDO-Im modify Cys151. This modification is suggested by another model to be important in NRF2 activation because it disrupts KEAP1-CUL3 association. Both discussed models suggest that newly synthesized NRF2 translocates into the nucleus [75,76].

Further, NRF2 dimerizes with the small MAF (sMAF) protein to form NRF2-sMAF heterodimers. sMAF proteins have a bZIP domain which is similar to the one present in NRF2. NRF2-sMAF heterodimer formation ensures binding of NRF2 to ARE due to the high concentration of this heterodimer in the nucleus [78,79].

NRF2 binds to cis-regulatory ARE (5′-RTGACnnnGC-3′) to control the basal and inducible expression of antioxidant and detoxifying genes under stressed conditions caused by xenobiotics, metals, and UV irradiation [80,81,82]. ARE sequences are present in the promoters of different genes such as glutamate-cysteine ligase catalytic—(*GCLC*) and modifier subunit (*GCLM*), NAD(P)H quinone oxidoreductase 1 (*NQO1*), heme-oxygenase-1 (*HMOX1*), sulfiredoxin1 (*SRXN1*), glutathione S-transferase (*GST*), thioredoxin (*TXN*), glutathione reductase (*GSR*), superoxide dismutase 1 (*SOD1*), multidrug resistance-associated proteins (*MRPs*), and UDP-glucuronosyltransferase (*UGT*) [83]. The presence of ARE in the *HMOX1* promoter allows for the NRF2 mediated synthesis of the corresponding protein, heme oxygenase 1 (HO-1), the enzyme that catalyzes the first and rate limiting step in heme degradation [84]. 

Commonly presented in an overly simplistic fashion, the multilevel, fine regulation involved in NRF2 mediated transcription must be explored in a broad, yet well-controlled scenario.

In the absence of oxidative stress, heterodimers, consisting of MAF proteins and the transcription repressor BACH1 (BTB and CNC homology 1), a natural NRF2 competitor and molecular sensor of intracellular heme, occupy AREs [85]. Upon stress induction (as shown with exposure of HepG2 cells to tert-butyl hydroquinone), BACH1 becomes phosphorylated at Tyr 486 and is exported from the nucleus, allowing the incoming NRF2 free access to the ARE [86]. When a favorable redox balance is achieved by NRF2, newly synthesized BACH1 enters the nucleus and represses ARE elements. Most forms of oxidative stress elicit heme release from hemoproteins, leading to more oxidative stress. Increased levels of free heme promote binding of Bach1 and Fbxo22 ubiquitin ligase and consequential proteasome-dependent degradation of Bach1 [87]. This regulatory model was explored in-depth using the experimental model with a specific genetic background, with respect to Hmox1.

When comparing genetically engineered KP mice (Kras^LSL-G12D/+^; p53^flox/flox^) with KPK mice (KP mice with an additional to Keap1 deletion), Lignitto et al. observed that, although the size of tumors developed did not differ, KPK mice develop high number of lung metastases, associated with both, Nrf2 and Bach1 signatures [53]. The underlying mechanisms for increase of the stronger, Bach 1 signature, was consequential to a decreased level of Bach1 degradation due to: (a) Nrf2-dependent increase of heme oxygenase 1 (b) increased rate of heme degradation; (c) decreased heme-dependent binding of Bach1 and Fbxo22 ubiquitin ligase; (d) decreased rate of Bach1 proteasome-dependent degradation. Finally, the authors were able to show that lack of Fbxo22 strongly activates the Bach1-prometastatic transcriptional program, in KPK cells. In this complex scenario, based on various interactions, Bach1 was shown as a promoter, while Hmox1 was shown as an inhibitor of metastases formation. The prognostic significance of strong Bach1 signature was further shown in LUAD (Lung Adenocarcinoma) dataset, where its high levels were associated with increased metastatic potential and poor survival [53].

In a similar experimental model, an increased Bach1 associated metastatic potential was achieved through reduction of free heme levels consequential to application of antioxidants (N-acetyl cysteine (NAC) and vitamin E). Experimentally was shown that Bach 1 contributes to glycolysis and increased production of ATP through its binding to Hexokinase 2 (*Hex 2*) and Glyceraldehyde-3-Phosphate Dehydrogenase (*Gapdh*) promoters [88].

All these data indicate the complexity of signaling pathways which include NRF2, its targets and its competitors, and are developed in a specific cellular background. They further indicate that the roles of NRF2 in cancer must be considered from many aspects, and that additional data are necessary to clarify whether selective targeting of NRF2 has the potential to be implemented in the field of cancer therapy.

## 3. Regulation of NRF2 Expression in Cancer

The *NFE2L2* gene, resides on a long arm of human chromosome 2 (2q31.2). As already explained, the level of the cellular NRF2 protein relies on at least dual regulation (protein degradation level/transcriptional activity of the *NRF2* gene). While there are numerous scientific articles dedicated to the function of the NRF2 protein which are primarily related to its master regulatory involvement in the maintenance of the cellular redox status, there are only a few papers dealing with the transcriptional regulation of the *NRF2*. They are closely related to the structure of the *NRF2* promoter, including the presence of specific single nucleotide polymorphisms (SNPs).

**Figure 2:**
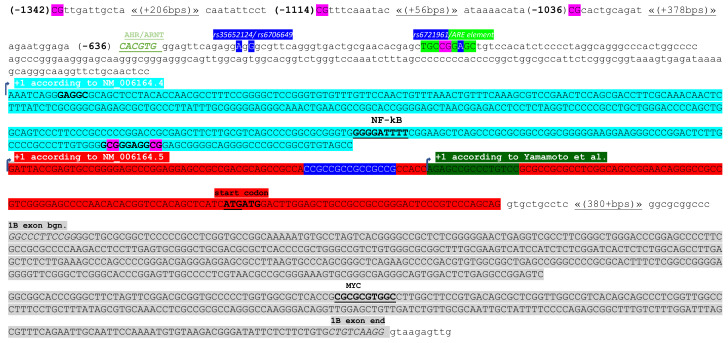
*NRF2* promoter, presented from −1342. The most current TSS is presented in red highlight (NM_006164.5), and the previous version is presented in turquois (NM_006164.4). The TSS described by Yamamoto is shown in green highlight [89]. Differentially methylated CG spots are presented in pink, including the one in the ARE element. Transcription factor binding sites for AHR/ARNT (Aryl Hydrocarbon Receptor/Aryl Hydrocarbon Receptor Nuclear Translocator), NF-κB, and MYC are underlined. ATG: first coding triplet. Polymorphism are presented in a blue highlight.

The first polymorphisms (three SNPs and one repetitive triplet) in the *NRF2* promoter were discovered in 2004, by Yamamoto and collaborators [89]. Based on then known gDNA sequence, these variations were considered to be positioned at −686, −684, −650 and −20 to −6 (CCG)n upstream from the transcription start site (TSS). However, based on our knowledge of the current *NRF2* genomic DNA (NC_000002.12) and corresponding mRNAs, these SNPs are 68 bps closer to the TSS, at −618, −616, and −582. The variable number of CCG triplets is actually present in the non-coding part of the first *NRF2* exon (Figure 2, white letters, blue highlight). The three SNPs originally discovered in 2004 were deposited in the SNP database as rs35652124 (T > A/T > C/T > G), rs6706649 (C > T), and rs6721961 (T > A/T > C/T > G). The functional role of the SNP rs6721961 was shown in 2007, by Marzec and collaborators [90].

It is now well accepted that human *NRF2* promoter contains ARE that allows for autoregulation of the *NRF2* transcription. Using a model of oxidant-induced acute lung injury (ALI), Marzec et al. [90] have shown that rs6721961 (in the original study numbered as −617, with respect to the TSS) resides in the ARE element of the *NRF2* promoter (TGCCGGC/AGC; Figure 2, shown in green highlight). They showed that patients who were heterozygous, A/C, had a significantly higher risk for developing ALI after major trauma compared with patients with the A/A genotype. The underlying molecular mechanism involved was shown to be a less efficient binding of NRF2 to the polymorphic allele [90].

In mice, *Nrf2* transcription may be also activated through binding of aryl hydrocarbon receptor (AHR) to the three Xenobiotic Response Elements (XRE) present in the *Nrf2* promoter region [91]. Although the existence of the five XRE elements are predicted to reside in the human *NRF2* promoter [92], experimental data for confirming AHR binding to these elements cannot be found. Still, based on the transcriptional regulation of some other genes included in the NRF2 signaling cascade, one may hypothesize that binding occurs. In some experimental scenarios, *NQO1*—a bona fide target of NRF2 [93] was shown to be highly expressed at the intersection of the AHR and NRF2 transcriptional signatures [94]. Still, instead of activating *NQO*1 transcription indirectly, through NRF2, AHR can activate *NQO1* directly, due to the presence of XRE elements in the *NQO1* promoter [95].

In addition to the existence of the autoregulatory loop mediated by NRF2 binding to the ARE element present in its own promoter, several other transcription factors are involved in the transcriptional regulation of *NRF2* activity. Based on data published so far, cell-type specific regulation of *NRF2* transcriptional activity is not in question. In 2012, Rushworth [96] and collaborators have shown that the NF-κB heterodimer P50/P65 specifically binds to the *NRF2* promoter, induces its transcriptional activity, and leads to enhanced activation of NRF2-dependent antioxidant defense responses. When activating lymphoblastoid cell line GM12891 by TNF-α, the authors were able to show that the transcriptional start site of the *NRF2*, which overlaps with sites of RNA POL II binding, is under the transcriptional control of the NF-κB subunit P65 (REL-A). We were modeling the sequence covered by primers published [96] through JASPAR database and were able to find the strong P65 binding site, GGGGATTTT (score: 10.559007), which starts at the position −115, according to NM_006164.5 and is −266 bps distant from the first coding triplet (Figure 2, in turquoise highlight; bold, underlined).

In 2001, Hattori et al. have shown using the non-cancer (vascular smooth muscle cells), murine in vitro model, that HNE has an impact on NF-κB dependent transcriptional activation of inducible NO synthase (iNOS), through the inhibitory effect on proteolytic degradation of the cytosolic NF-κB inhibitory protein, IκBα. Considering the cell-type specific regulation of NRF2 activity, it would be of interest to investigate the effect of HNE on NRF2 transcription in leukemic cells. As well, HNE is known to suppress the growth of leukemic cells while enhancing the growth of normal human lymphocytes [97,98].

In 2004, Bae et al. have shown that Breast Cancer Type 1 Susceptibility Protein (BRCA1) has stimulative effect on ARE-dependent transcription specifically associated with *NRF2*, in vitro [99]. In 2011, Kang et al. [100] found that immortalized, non-tumorigenic breast cell line MCF 10A with silenced BRCA1 has decreased expression of NRF2, while, on the other hand, exposure to benzo[a]pyrene (BaP) increases the NRF2 protein level, while no increase in *NRF2* mRNA was observed. It has been shown that BRCA1 has an ability to bind to non-B DNA structures, especially to cruciform and quadruplex DNA structures [101]. However, strong BRCA1 binding to one XRE element was demonstrated [100]. Although BRCA1 does not bind to any preferential DNA sequence, it physically binds to AHR/ARNT and enhances xenobiotic stress-induced gene activity [102], including *NRF2*. In that specific scenario, using the JASPAR program, we were able to map several potential BRCA1/AHR/ARNT binding DNA sequences. However, only one, positioned between −636 and −631 (CACGTG; Figure 2, green letters, bold, underlined), had a prediction score higher than 10 (10.3510685). When exploring that short sequence more carefully, we became aware that part of it may be deleted or duplicated when the rs1690906874 is present. Based on data deposited in the SNP database, this polymorphism is very rare and there is no proof for its clinical relevance.

Exposure to nicotine and acrolein, which are the risk factors for the occurrence of head and neck squamous cell carcinomas (HNSSCs), was shown to increase both MYC Proto-Oncogene Protein (c-MYC) and BRAF [103]. However, only c-MYC can bind the *NRF2* promoter and activate the *NRF2* transcription. Based on the published primer sequences [103], the c-MYC binding site is in the *NRF2* exon 1B, between +1081 and +1090 (binding sequence: CGCGCGTGGC, score: 10.485164, according to our own modeling through JASPAR, Figure 2). In HNSCC tumors, NRF2 was shown to be bound to ARE elements of 6-phosphogluconate dehydrogenase (PGD) and transketolase (TKT), leading to their enhanced expression. The activity of this signaling pathway, directed by c-MYC and NRF2, seems to be highly prognostic for the HNSCC patients [103]. The overall survival analysis revealed that the patients in the NRF2/G6PD/TKT-high group (N = 102) had significantly (*p* = 0.005) worse survival outcomes when compared to NRF2/G6PD/TKT-low patients’ group (N = 113).

This is in agreement with DeNicola’s study which previously showed high transcriptional activity of *NRF2* in cells harboring K-*RAS* mutation. The study also showed that *NRF2* transcription in this molecular scenario highly depends on c-MYC, JUN and FRA1 [104]. Few years later, the same authors showed that NRF2 positively regulates expression of serine biosynthetic enzymes and contributes to the poor prognosis of patients suffering from non-small cell lung cancer (NSCLC) [105].

Currently there are eight deposited *NRF2* mRNAs (Table 1). These transcription variants (TVs) have identical exons 2 and 5. However, they differ with respect to TSS (position of exon 1, which we call 1A and 1B), inclusion of exon 3 (excluded in TV6) and exon 4 (shorter in TV3). Here we presented the promoter structure (Figure 2) with respect to transcript variants 1, 6 and 7, containing exon 1A (Table 1). The rare NRF2 splices mutations, demonstrated as loss of exon 2, exist in HNSCC (1.5%; 6/403) and NSCLC (3.3%; 16/481) [106]. The Table 1. should be helpful for construction of appropriate *NRF2* primers for end-point PCR based exploration of *NRF2* splice variants, and especially for selection of appropriate TaqMan assays.

These sequences have been updated very recently and for that reason some discrepancies between data shown in Table 1 and mRNAs start sites presented in the *NRF2* genomic sequence (NCBI Reference Sequence: NC_000002.12; Homo sapiens chromosome 2, GRCh38.p13 Primary Assembly; REGION: complement (177230303…177265131), currently exist. The difference makes 404 nucleotides, with respect to the TSS of variants 1, 6 and 7, containing exon 1A. That nucleotide sequence is shown on Figure 2, in turquoise. In 2014, when NM_006164.4 was considered to be the TV1, Khor et al. [107], demonstrated differential methylation status of three CpG spots residing at -1342, -1114 and -1036 in human prostate cancer samples (Figure 2, in pink). When comparing nine benign prostate hyperplasia (BPH) tissues with seven androgen-stimulated prostate cancers (AS-CaP) and 11 androgen-deprivation therapy recurrent prostate cancers (ADTRCaP), increased, differentially methylated status of the cancer tissue (AS-CaP and ADTRCaP; 25.3% and 43.6% methylation, respectively) was discovered. In BPH, there was only 19.6% methylated spots. It was additionally shown that these differentially methylated CpGs, which are enriched with H3K9m3, are sensitive to both, the DNA demethylation agent 5-Azacytidine and Trichostatin A (TSA), an inhibitor of histone deacetylases [107]. We have explored the *NRF2* promoter with respect to differentially methylated region and noted that, among 213 predicted specific CpG sites one resides in the *NRF2* ARE element (Figure 2, CG in pink).

The status of DNA methylation seems to be connected to the HNE-mediated occurrence of DNA adducts, indicating a functional connection between lipid peroxidation and epigenetic regulation. Namely, exposure of the wild-type TP53 lymphoblastoid cell line TK-6 to HNE results in an increased rate of G to T transversions, AG**G** to AG**T**, at TP53 codon 249 [108]. This finding was later confirmed, and some new HNE-DNA adducts were discovered after exposure of TP53 DNA fragments corresponding to exons 5, 7, and 8 to HNE [24]. The same article has documented a preferential binding of HNE to specific DNA sequences: -CGGAGG*C-/-AGG*CGC- (corresponding to codons 249 and 174), -CAGG*A- and -GAGG*AA (corresponding to codon 286) (*: adducted guanine base). Although it is well known that methylated cytosines make preferential spot for carcinogen–adduct formation [109], in the described TP53 based model, the HNE binding at codons 249 and 174 was not affected by C5 cytosine methylation [24]. In the promoter of *NRF2*, the -GAGGC- sequences start at only −396 and −37 bps upstream of the NM_006164.5 TSS. According to Khor’s map [107], the second sequence, closer to the TSS, is part of the nucleotide stretch which contains two (out of 213) specific CpG sites: GCGGGAGGCG, shown to be methylated (Figure 2). The possible biological consequence, related to the high probability of HNE-DNA adducts occurrence in this specific part of the *NRF2* promoter region remains to be further investigated.

There is yet one more, very powerful level of NRF2 epigenetic regulation. Micro RNAs (miRNA) are a type of non-coding, 20–22 nucleotides long RNAs involved in fine regulation of expression of numerous genes. It was originally estimated that human miRNAs have a selective pressure for pairing with more than 60% of protein coding genes [110]. Currently, there are 2654 human, mature, annotated miRNAs [111]. For this reason, they are crucial players involved in a plethora of physiological and pathophysiological processes [112], including those related to NRF2 and KEAP1 (Table 2).

There is strong experimental evidence that numerous miRNAs regulate NRF2 and/or KEAP1 post-transcriptionally, leading to a decrease of corresponding proteins in various cell types.

The influence of HNE on miRNAs have been shown in different experimental models (retinal pigment epithelium [113] and retinal capillary endothelial cells [114]), as well as in the experimental model of human leukemia. Therefore, we may hypothesize that this particular product of lipid peroxidation has a pleiotropic effect on NRF2, irrespective of the cell origin (malignant vs. non-malignant), but in dependence of different modes of action [115].

Since an active NRF2 may modulate cellular metabolism and exert an oncogenic potential, targeting NRF2 by mi-RNA seems an attractive therapeutic approach. By using luciferase assay and HeLa cells, Yamamoto et al. have discovered that four miRNAs: miR-507 (Xq27.3), miR-634 (17q24.2), miR-450a (Xq26.3) and miR-129-5p (7q32.1) can bind to 3′ untranslated region (3′UTR) of the *NRF2* mRNA. Consequentially, these miRNAs contributed to the reduction of the NRF2 protein, which was associated with increased HeLa sensitivity to H_2_O_2_ and cisplatin. By analyzing the level of these four miRNAs in 30 samples of primary esophageal squamous cell carcinoma, they have detected more than 50% decrease of miR-507 (9/30 samples), miR-634 (12/30 samples), miR-450a (2/30 samples) and miR-129-5p (18/30 samples) in tumors compared to surrounding non-tumor tissues [116]. When focusing on miR-507, the authors were able to show the beneficial effect of its exogenous application to animals bearing A549 originating tumors (NRF2 overexpressing/KEAP1 mutated), that was demonstrated through inhibition of the tumor growth.

Narasimhan et al. have demonstrated that overexpression of each of the following miRNAs: miR-144 (17q11.2), miR-153 (2q35), miR-27a (19p13.12), miR-142-5p (17q22) in neuroblastoma cell line SH-SY5Y reduces the level of NRF2 protein. Additional experiments based on luciferase activity assays have shown that these miRNAs indeed bind to *NRF2* 3′UTR but are unable to bind it if the seed 3′UTR region is mutated [117].

Another miRNA that binds to the 3′UTR of NRF2 mRNA is miR-28 (3q28), as shown in the human embryonic kidney cell line HEK293. Transfection of miR-28 into the MCF-7 breast cancer cell line reduces both NRF2 mRNA and the protein. Sequential experiments have shown the involvement of miR-28 in accelerated degradation of NRF2 transcript and a decrease in the stability of NRF2 protein, independent of KEAP1 [118].

Singh et al. have explored the possible connection between the estrogen treatment of female ACI rats and the levels of miR-93 in their mammary tissues. They have discovered elevated levels of miR-93 accompanied by reduced levels of NRF2 protein. Additional experiments have shown that overexpression of miR-93 reduces the level of NRF2 protein. Unfortunately, the level of NRF2 mRNA before and after transfection of miR-93 was not determined [119].

This area of research on NRF2 epi-regulation will certainly prosper in the future, fruitfully combined with exploration of miRNAs able to post-transcriptionally regulate KEAP1.

Since KEAP1 is the major regulator of NRF2 protein subcellular localization and stability, miRNAs that effect KEAP1 invariably influence NRF2.

MicroRNA 200-a (1p36.22) has been shown to bind to 3′ UTR of KEAP1 mRNA, resulting in decreased KEAP1 mRNA stability, and reduced KEAP1 protein level in two breast cancer cell lines: MDA-MB-231 and Hs578T. Expectedly, overexpression of miRNA-200a resulted in elevated NRF2 protein level, as shown in whole-cell lysates and nuclear extracts [120]. Van Jaarsveld et al. investigated the role of miR-141 (12p13.31) in ovarian cancer cell line A2780. They have discovered that overexpression of miR-141 significantly reduces both, KEAP1 mRNA and the corresponding protein. Additional experiments, including luciferase assay, provided further evidence that miR-141 binds to KEAP1 3′UTR. Conversely, mutation of the binding site in KEAP1 3′ UTR abolishes the effect [121]. Similarly, overexpression of miR-7 (9q21.32) in neuroblastoma cell line SH-SY5Y reduces the level of KEAP1 protein and leads to an increase of NRF2 protein in the nucleus of these cells. However, it does not significantly affect the level of KEAP1 mRNA [122].

In HeLa cell line, miR-432-3p (14q32.2) was shown to be a binding partner for KEAP1 mRNA. This binding results in reduced stability and amount of KEAP1 mRNA, as well as reduced KEAP1 protein level [123].

In the end, the role of miRNAs which target NRF2/KEAP1 axis in cancer cells is most likely dependent on the broader cellular context. Overexpression of NRF2 in certain cancers can cause resistance to chemotherapeutic agents, while expression of NRF2 targeting miRNAs may undermine this process. However, since NRF2 plays a crucial role in protection of cells from oxidative stress, higher expression of these miRNA may suppress NRF2 and lead to an elevated level of cellular oxidative stress. Same, albeit reverse, argument can be applied to miRNA that target KEAP1.

## 4. Multilevel Models of Communication between NRF2 and TP53

In 2006, Faraonio et al. showed that WT TP53 suppresses the activity of ARE elements through direct interaction [124]. New data are showing that, in lung cancer, *NRF2* transcription depends on the TP53 mutational status. In a TP53 WT (wild type) lung cancer cells, *NRF2* transcription is decreased due to the WT TP53 related decreased binding of SP1 to *NRF2* promoter. This effect is absent in tumors harboring the MT TP53. As a consequence, SP1 strongly binds to *NRF2* promoter and activates its transcription, with consequential increased transcription rate of *HMOX1*, *BCL2* and *BCL-XL*. This proteo-transcript profile is associated with resistance to cisplatin, in NSCLC lung cancer. The increased transcription rate of *NRF2* was shown to be strongly associated (*p* = 0.013) with worse overall survival (OS) and recurrence free survival (RFS), *p* = 0.022 [125]. A breast cancer cell model was used to show the binding of MT TP53 and NRF2 proteins through TP53 amino acids 98–128, which are the part of the TP53 DMA binding domain (DBD). The results obtained indicated a synchronous way of acting through which, during induced oxidative stress, mutant TP53 requires NRF2 for binding to ARE sequences, and increases its binding to ARE of basally active NRF2 targets (thioredoxin system: thioredoxin (TXN) and thioredoxin reductase (TXNRD1); 26S proteasome subunit gene promoters), leading to proteasomal degradation of tumor-suppressor proteins and acting pro-oncogenic [126,127]. Consequentially, less NRF2 is bound to ARE of NRF2 inducible genes; those whose expression is low in the absence of oxidative stress (*ABCC3* (ATP-Binding Cassette Sub-Family C Member 3), and *HMOX1*) [127].

All the described interactions should be considered in a specific time windows of cellular exposure to various stressors, keeping in mind the existence of multilevel modes of communication among cellular signaling pathways. This is especially important in the context of recent research of Hiemstra et al. [128], who were exploring both, NRF2-mediated- and TP53-mediated cellular response to oxidative stress inducer diethyl maleate (DEM) and DNA damage inducer and therapeutic-etoposide. They were able to show that both signaling pathways are activated in these scenarios, in a concentration-dependent fashion. They were also able to show a strong activation of *P21* (cyclin dependent kinase inhibitor 1A, *CDKN1A*), a bona fide TP53 target, as a consequence of KEAP1 knockdown. Vice versa, knockdown of TP53 resulted in slight, but significant decrease of the NRF2 target, Sulfiredoxin 1. These facts, in addition to already known phenomena involved in preservation of balance between TP53 and NRF2 signaling pathways [129,130], strongly point to the existing, yet highly complex communication between these two signaling pathways which are relevant for all aspects of cellular life, including the response to therapy.

## 5. The Role of NRF2 in Cancer Stem Cells

NRF2 is the main regulator of ROS level and of the cellular antioxidant profile targeting numerous genes already mentioned, together with WNT and NOTCH, which are important for self-renewal. It is known that NRF2 maintains cancer stem cells (CSCs) in an undifferentiated state. NRF2 overexpression is crucial for CSCs to maintain stemness [131]. NRF2 knockdown significantly reduces stem markers in CSCs and induces their differentiation, as shown in glioma model [132].

Silencing of NRF2 also suppressed CSCs markers in breast cancer model [133] in ALDH-high ovarian cancer cells [134], and sorafenib-resistant hepatocellular carcinoma cells [135]. Additionally, NRF2 binds directly to upstream regions of pluripotency genes (*OCT4* and *NANOG*) to promote their expression [136]. 

Cancer stem cells have been studied more excessively in recent years, and different authors have reported that certain CSCs related phenomena involve NRF2. Latest studies demonstrated that breast cancer stem cells (BCSCs) exhibit plasticity enabling them to transition between two phenotypic states: a proliferative epithelial-like (E) state with high expression of aldehyde dehydrogenase (ALDH), and a quiescent, invasive mesenchymal-like (M) state, characterized with CD24^−^CD44^+^ expression, which is similar to epithelial-to-mesenchymal transition (EMT). Luo et al. demonstrated that E CSCs have low ROS levels, while M CSCs cells have high ROS levels. Moreover, E-BCSCs display a strong NRF2-mediated antioxidant response [137]. Fiorillo et al. described new tumor cells isolated in their lab which they named energetic cancer stem cells (e-CSCs) which are, among other properties, characterized by high NRF2-mediated antioxidant response signature [138]. 

Kipp et al. revealed spatiotemporal patterns of expression of HIF and NRF2 during spheroid formation, i.e., the cancer cell growth in the 3-D culture system characterized by floating spheres with features of CSCs. On the first day of spheroid formation, these factors were activated and thereafter became repressed. However, they were reactivated again, after a week within the spheroid core. The NRF2 inducer stimulated proliferating differentiated spheroids, while HIF inducer triggered a highly resistant quiescent phenotype [139].

Additionally, many new signaling pathways have been linked to NRF2 and CSCs formation and maintenance. Kim et al. recently showed a novel pathway by which NRF2 may promote favorable conditions for CSCs maintenance. Breast cancer stem-like cells were shown to have an elevated production of reduced GSH maintained by upregulation of NRF2 target gene, *GCLC*, which lowered ROS levels. This, in turn, induced the activation of AMP-activated protein kinase and FoxO3a through phosphorylation. FoxO3a binds to the *Bmi-1* promoter which contributes to the self-renewal activity and tumorigenesis [140]. Sun et al. demonstrated another signaling pathway involving NRF2 and CSCs enrichment in hepatocellular carcinoma. They showed that xanthine oxidoreductase inhibits liver CSC survival and tumor promotion through interaction with ubiquitin-specific peptidase 15 which promotes deubiquitylation of KEAP1 leading to NRF2 degradation [141]. Wang et al. reported that autophagy and NRF2 are the two most important factors for ovarian cancer spheroid cells survival. Autophagy is critical for quiescent ovarian cancer stem cells to re-enter the cell cycle and optimal ROS levels increase self-renewal marker NOTCH1 [142].

Recent studies revealed that arsenic exposure may induce NRF2 dependent generation of cancer stem-like cells [143,144,145]. Furthermore, Bi et al. showed that arsenic-induced metabolic reprogramming is dependent on NRF2 and HIF1α. NRF2 has been demonstrated crucial in the regulation of metabolic shift from the tricarboxylic acid (TCA) cycle to glycolysis during CSCs generation [146].

Another recent report showed that NRF2 plays also important role in iron homeostasis as it controls the expression of ferritin light chain (FTL), ferritin heavy chain (FTH), and ferroportin (FPN). Consequently, ferroptosis, a non-apoptotic cell death dependent on intracellular iron and accumulation of lipid peroxidation products, especially HNE, may cause the death of the CSCs [147,148]. Čipak et al. found that HNE itself can be cytotoxic for the BCSCs in vitro, especially if the cells were seeded on the oxidized collagen matrix resembling the in vivo situation of inflammation and/or radio/chemotherapy [149]. A recent study, aimed to reveal the impact of oxidative stress on CSCs, revealed that low levels of HNE can increase differentiation markers in CSCs, while higher levels increased GSH, NRF2, and EMT markers [150]. That also resembles effects of HNE on human osteosarcoma cells for which HNE can exert cytotoxic effects proportional to their level of differentiation [151].

NRF2 is also important for CSCs drug resistance acquisition. The NRF2-silenced mammospheres that did not develop anticancer drug resistance demonstrated increased cell death and delayed growth [152]. Goto et al. recently showed that increased expression of ATP Binding Cassette Subfamily B Member 1 (ABCB1, commonly known as MDR1) and NRF2 are associated with doxorubicin resistance of CD44^+^CD133^+^ cells, but not in case of CD44^+^ and CD44^+^CD133^−^ cells [153]. Noman et al. recently reported that high expression of Sonic hedgehog and NRF2 correlates with induced stem cell-like characteristics and contributes to chemoresistance of HNSCC cells [154]. A very recent study showed that dexamethasone can induce chemosensitization of CSCs through reduced expression of NRF2 and consequently increased levels of ROS, but only after the treatment with gemcitabine and 5-fluorouracil [155]. A natural product, chestnut leaf extract, has been shown to increase the chemosensitivity of breast cancer stem cells to paclitaxel through the suppression of NRF2 [156]. Increased expression of NRF2 is responsible for multidrug resistance in head and neck squamous cell carcinoma stem cells positive for CD133 marker [157], as well as in ovarian cancer stem cells [158]. Achuthan et al. revealed that some cancer cells escape drug-induced cell death after chemotherapy followed by a senescent state associated with relatively high levels of ROS [132]. After that, most of the cells underwent unstable multiplications followed by spontaneous cell death. However, some cells formed stable colonies, with stem cell-like aggressive phenotypes, and were characterized by high CD133 and Oct4 expression [159].

Furthermore, NRF2 seems to be an important factor in acquiring CSCs resistance to radiation therapy of breast cancer cells, [133,160] and glioblastoma [161]. Summary of the role of NRF2 in different cancer stem cell models is presented in the Table 3.

Although majority of the data linking NRF2 and CSCs were obtained in vitro, it seems that the inhibition of NRF2 in CSCs may be a promising option for cancer therapies which includes radiation sensitization.

## 6. NRF2 Mediated Polarization of Neutrophils and Macrophages in Cancer

Another important aspect of NRF2 in carcinogenesis is its involvement in regulation of signaling pathways in inflammatory and stromal cells. The NRF2 has a dual role in the regulation of inflammation in cancer, through which it may either promote or inhibit anticancer immunity. Both pro-tumor and anti-tumor roles of NRF2 have been recently reviewed in several excellent papers [162,163,164].

Neutrophils (a major class of polymorphonuclear leukocytes) are the most abundant leukocytes in circulation and are among the first line of immune defense against invading pathogens. Neutrophils are also the first responders to the tissue damage. Together with monocytes, neutrophils infiltrate tumor tissue where they may have diverse roles, either in promoting or inhibiting tumor growth [165,166,167,168].

Tumor associated neutrophils (TANs) and macrophages (TAMs) are exposed to a plethora of factors present in the tumor microenvironment that do not originate only from tumor cells, but also from other stromal cells. Signals received from tumor microenvironment can induce polarization of immune cells into functionally distinct TANs and TAMs populations [169,170,171]. The TGF-β induces N2 population of TANs with pro-tumor phenotype [172], while type I interferons (IFNs) promote polarization of TANs to N1 population with anti-tumor phenotype [173]. Similarly, IFN-γ promotes polarization of macrophages to M1 anti-tumor phenotype, while the presence of cytokines, such as interleukin (IL)-4, IL-10 and IL-13 stimulate M2 TAMs with pro-tumor phenotype [174]. In response to adequate stimuli, TANs and TAMs produce and release ROS. However, different TAN and TAM populations have distinct ROS profiles. Activated M1 generate higher levels of ROS and are more resistant to alterations in cellular redox status than M2 [175]. Recent studies suggested similar for neutrophils, where elevated production of MPO-derived ROS was observed for N1 [176] as well as neutrophil NADPH-oxidase-derived ROS mediated tumor growth inhibition [177]. Thus, inflammatory ROS can interact with redox sensitive molecules and promote redox signaling pathways, including those associated with NRF2 in cancer and in inflammation [164]. As mentioned before, impaired redox homeostasis and elevated ROS can affect the structure and function of major macromolecules [178], among which polyunsaturated fatty acids are particularly susceptible to ROS-induced damage leading to the formation of reactive aldehydes, including bioactive HNE that can modulate various signaling pathways, both under physiological and pathological processes [179,180,181,182,183], as previously discussed.

Furthermore, lactate, produced via aerobic glycolysis by tumor cells, upregulates macrophage intracellular ROS and triggers NRF2 activation. As a consequence, there is a polarization of macrophages to the M2 phenotype [184]. In turn, M2 derived VEGF promotes activation of NRF2 in neighboring tumor cells, supporting cancer cell epithelial-mesenchymal transition [184]. In addition, Kobayashi et al. reported that activation of Nrf2 inhibits *Il-6*, *Il-1b*, *Il-1a*, *Il12b* and *Nos2* expression in M1, while it has no effect on *Tnf* and *Irf1* [185]. 

Furthermore, as shown in lupus, activation of Nrf2 in macrophages downregulates IFN receptor and IFN-stimulated gene expression, while its inhibition has the opposite effect thus interfering with macrophage polarization [186]. 

ROS are also necessary for IL-4 induced Stat3 activation during M2 polarization, while the M2 polarization can be prevented by the SOD mimetic [175]. A very recent study demonstrated that oncoprotein multiple copies in T-cell malignancy-1 (MCT-1) stabilizes NRF2 to transcriptionally induce *SOD2* in triple-negative breast cancer (TNBC) cells where SOD2 acts as prooxidant peroxidase and is involved in mitochondrial ROS production, TNBC cell invasion and IL-6 secretion promoted by MCT-1 [187]. Silencing of SOD2 promotes antitumor effects of M1 and prevents polarization of M2 affecting tumor cell progression [187]. Activation of the Nrf2 pathway, and upregulation of its target genes/proteins can further impact macrophage polarization. For example, in non-tumorous models, TRX-1 was reported to promote M2 phenotype [188], while GSH promotes M1 phenotype [189]. As recently reviewed, elevated TRX rise associated with tumor progression and poor patient outcome [164]. The upregulation of Nrf2 target, heme oxygenase 1, reduces the M1 polarization in breast cancer TAMs, while inhibition of HO-1 diminishes that effect [190]. While the overexpression of NRF2 targets contributes to macrophages plasticity in cancer directly, it is challenging to hypothesize about NRF2 acting indirectly, through extracellular tumor-derived vesicles [191].

**Figure 3:**
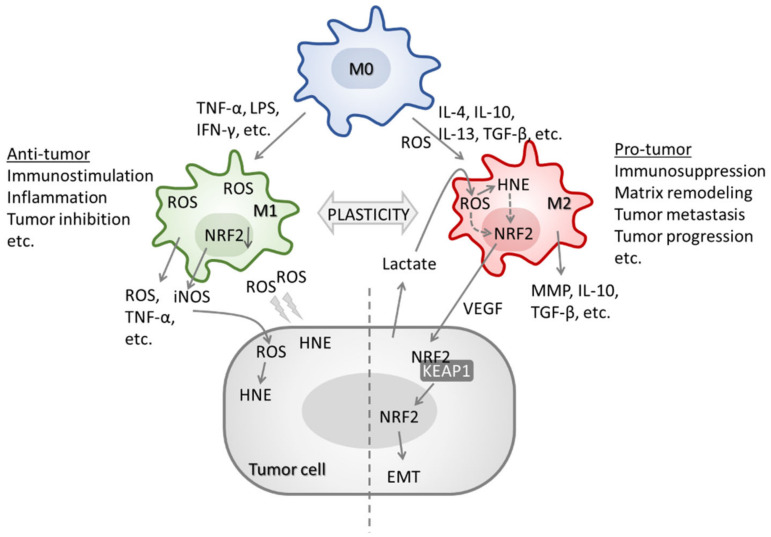
The involvement of cytokines, ROS, HNE, and NRF2 in TAM polarization. In response to TNF-α, LPS or IFN-γ, TAMs are differentiated into M1 phenotype. M1 TAMs express high levels of iNOS, ROS, and TNF-α promoting HNE, immunostimulation, inflammation, and inhibiting tumor growth. In contrast, in response to IL-4, IL-10, IL-13 or TGF-β, TAMs are polarized to M2 phenotype. Although M2 TAMs have lower ROS compared to M1, ROS are essential for NRF2 activation and M2 polarization. M2 TAMs produce TGF-β, MMP, IL-10, and VEGF promoting matrix remodeling, EMT, tumor growth, and metastasis.

The involvement of Nrf2 in macrophage polarization was also demonstrated in acute respiratory distress syndrome model, where silencing of Nrf2 upregulated iNOS and IL-10 promoting M1 population, while polarization to M2 population was dependent on Nrf2 activation [192]. Interestingly, the studies with murine bone marrow-derived macrophages did not show any effect of Nrf2 activation on macrophage differentiation and maturation [185]. The TAM polarization to M1 and M2 phenotypes with involvement of cytokines, ROS, HNE, and NRF2 is illustrated on Figure 3.

Furthermore, various stimuli, such are granulocyte-macrophage colony-stimulating factor (GM-CSF) and IFN-γ, increase Nrf2 expression in neutrophils isolated from both wild type animals and KO animals for purinergic receptor, the P2RX1. The Nrf2 activation was accompanied by metabolic reprogramming and polarization to neutrophils [193]. However, the role of Nrf2 in polarization of neutrophils and macrophages is still underexplored and requires further attention.

Although the recruitment of neutrophils and macrophages might be essential for the destruction of cancer cells and debris clearance, polarization to N2 or M2 will promote tumor growth and metastasis. Hence the ability to control polarization or to reprogram N2 or M2 macrophages to N1 and M1, respectively, might impair tumor growth and improve prognosis. Thus, understanding the mechanisms of the immune cells’ polarization to pro-/anti-tumor population, could represent a major step in the management of cancer growth control and anti-cancer therapies.

## 7. NRF2 and Cancer Resistance to Therapies

As a master antioxidative transcription factor, NRF2 is considered crucial in preventing cancer by keeping the redox homeostasis in normal cells [194]. Its activation in non-transformed cells promotes transcription of phase II antioxidant genes [195], resulting in increased antioxidative defense of the cell. This is favorable defensive mechanism against molecular damages that may lead to malignant transformation. Thus, it is beneficial for the cell and for the host. Once the cell transformation occurred, activated NRF2 shows its “dark side” [196]. Activation of NRF2 in cancer cells also increases defensive protective mechanisms that help in survival of cancer cells, promote cancer progression and metastases as well as resistance to radiotherapy and chemotherapy [162]. All these are beneficial for the survival of cancer cells, but are not beneficial for the host. These activities are not necessarily related to the role of NRF2 as the master antioxidant transcription factor, but to its ability to influence various cellular processes associated with drug metabolism: excretion, energy metabolism, iron and amino acid metabolism, mitochondrial metabolism, autophagy, and proliferation. All these lead to protection of the tumor cells from the therapy applied and place the NRF2 on the chart of cancer hallmarks regulators [162]. When discussing the role of NRF2 in radioresistance, NRF2 inhibition affected radioresistant colon cancer cell lines, SW1463 and HT55 shifting the resistant line to sensitive one, while it did not affect radiosensitive cell lines [197]. Authors suggest that this effect is achieved by NRF2 activation of metabolic shift [197]. In support of this finding is the regulation of Programmed Death-Ligand 1 (PD-L1) by NRF2 in colon cancer tissues, where up-regulation of PD-L1 by NRF2 create axis for oxaliplatin resistance [198].

Nowadays, an increasing amount of evidence suggests the role of NRF2 in cancer cell resistance to chemotherapeutics with a different mechanism of action. In hepatocellular carcinoma cell line Huh-7, resistance to sorafenib is accompanied by increased levels of NRF2 and HO-1 [135]. Knock-down of NRF2 in these resistant cells resulted in the reduction of stemness markers, decrease in proliferation, migration, and interestingly, reduction in ABCB1, ABCC1, and ABCG2 drug transporters expression [135]. In colon cancer, NRF2 overexpression correlated with stage and grade of tumor [199]. The resistance of colon cancer cells to oxaliplatin could be avoided by the inhibition of NRF2-PD-L1 axis [198]. Interestingly, the gemcitabine resistance of pancreatic cancer cells is supported by NRF2 signaling. Gemcitabine sensitivity can be obtained by inhibition of NRF2 by PIK-75 [200], which is supported by knockdown of NRF2 by siRNA in pancreatic cancer cell lines MIAPaCa-2, AsPc-1, and Panc-28 cells [201].

NRF2 dysregulation due to mutations in the NRF2 pathway (*NRF2*, *KEAP1*, and *CUL3*) is present in more than 1/3 of HNSCC negative to human papillomavirus (HPV), while in HNSCC positive to HPV, the rate of mutation in NRF2 pathway was extremely low or none [202]. This major difference in the mutation status of *NRF2* is in correlation with better overall survival of HNSCC positive to HPV compared to stage-matched HNSCC negative to HPV [202]. In support of this finding is that the NRF2 gain-of-function in HNSCC patients causes radioresistance [203]. More so, NRF2 pathway gene profiling is beneficial for HNSCC patients, as it stratifies patients who can benefit from the adjuvant platinum-based chemotherapy [204]. NRF2 dysregulation/activation occurs also due to mutations in its repressor, KEAP1. In lung adenocarcinoma with *KRAS* mutations, inactivating mutations in *KEAP1* are often co-occurring with serine/threonine kinase *STK11 (LKB1)* [205,206]. Series of co-occurring mutations in these genes *(KRAS*, *LKB1*, and *KEAP1*) associated with NRF2 overexpression drive tumor progression by causing and supporting metabolic shift and reprogramming [207] resulting in metastases and cis-platinum resistance [205]. The survival and proliferation of these cells are highly dependent on NRF2 activity. They are known as “NRF2-addicted cells” [52,208]. The metabolic features of NRF2-addicted cancers rely on both direct NRF2 targets (metabolic enzymes involved in glutathione synthesis, the pentose phosphate pathway (PPP) and NADPH production), and indirect NRF2 targets, of which ATF4 regulated serine biosynthetic enzymes, (*PHGDH*—Phosphoglycerate Dehydrogenase, *PSAT1*—Phosphoserine Aminotransferase 1 and *SHMT2*—Serine Hydroxymethyltransferase 2) correlate with poor prognosis in NSCLC [105]. They are highly dependent on extracellular glutamate [209], due to the high activity of NRF2 targets, GCLC and GCLM. They use glutamate for the synthesis of glutathione, and on the other hand, they secrete it through the cystine/glutamate antiporter xCT (*SLC7A11*). This metabolic profile of NSCLCs makes them sensitive to glutaminase inhibition [206]. This potential therapeutic approach seems to be also promising in K-ras mutated pancreatic cancer [210]. NRF2 pathway is intertwining with other signaling pathways, thereby changes in the activity of interacting pathways modulate NRF2 support and enhance tumor resistance. Such an example is the E3 ligase NEDD-4, which regulates PTEN (tumor suppressor Phosphatase and TENsin homolog) which further regulates PI3K/AKT/mTOR pathway and affects AKT/NRF2/HMOX-1 axis [211]. This sequence of events up-regulates the oxidative defense system and causes resistance to temozolomide in glioblastoma.

Pleiotropic HNE is a KEAP1 binding molecule that contributes to the detachment of NRF2 from KEAP1. In that fashion, it indirectly activates the NRF2 signaling pathway.

## 8. Conclusions

While NRF2 is physiologically potent regulator of complex antioxidant capacities of normal cells, thus preventing harmful effects of ROS, in malignant cells the protective capability of NRF2 to sustain the redox balance is abused to support their proliferation related metabolic requirements. The research on the epigenetic regulation of NRF2 will certainly prosper in the future. Additionally, exploration of miRNAs that are able to target KEAP1 will ay improve our knowledge of the NRF2/KEAP1 axis in all aspects of cellular biology, and especially in the field of oxidative stress and cancer, including its therapy response. The research should be complemented by a better understanding of HNE. It is a known activator of the NRF2 signaling pathway, which acts as a pleiotropic factor highly involved in redox signaling and homeostasis maintenance, in both, normal and cancer cells.

In conclusion, the NRF2 pathway itself or by interactions with other signaling pathways is highly important for tumor resistance to chemo-, radio- and antibody-based therapies. Therefore, it is an attractive target for advanced, highly selective cancer therapies.

## Figures and Tables

**Table 1:** Current NRF2 messenger RNAs deposited in the NCBI database (https://www.ncbi.nlm.nih.gov/gene/4780; accessed on 7 January 2022.

NRF2 Transcript Variants (TVs)	Exons Included	Exon 1A	Exon 1B	Exon 2	Exon 3	Exon 4	Exon 5
**1** **NM_006164.5**	**1..196, ** **30457..3072,** **31389..31478,** **32145..32336,** **32720..34425**	**196**		**267**	**90**	**192**	**1705**
**2 NM_001145412.3**	**597..1325, ** **30457..30723,** **31389..31478,** **32145..32336,** **32720..34425**		**729**	**267**	**90**	**192**	**1705**
**3 NM_001145413.3**	**597..1325, ** **30457..30723,** **31389..31478,** **32166..32336,** **32720..34425**		**729**	**267**	**90**	**192**	**1705**
**4 NM_001313900.1**	**597..1199, ** **30457..30723,** **31389..31478,** **32145..32336,** **32720..34425**		**603**	**267**	**90**	**192**	**1705**
**5 NM_001313901.1**	**597..1291, ** **30457..30723,** **31389..31478,** **32145..32336,** **32720..34425**		**695**	**267**	**90**	**192**	**1705**
**6 NM_001313902.1**	**1..196, ** **30457..30723,** **32145..32336,** **32720..34425**	**196**		**267**		**192**	**1705**
**7 NM_001313903.1**	**1..196, ** **30457..30504,** **31389..31478,** **32145..32336,** **32720..34425**	**196**		**267**	**90**	**192**	**1705**
**8 NM_001313904.1**	**597..1325, ** **30457..30652,** **31389..31478,** **32145..32336,** **32720..34425**		**729**	**267**	**90**	**192**	**1705**

**Table 2:** Human micro RNAs shown to interact with NRF2 and KEAP1, consequentially influencing the NRF2 signaling pathway. They are presented with respect to their chromosomal location and experimental/in silico seed sequences. Their binding sites are presented with respect to the beginning of the target gene sequence on corresponding chromosomes.

Hsa-miR	Locus	Mature miRNA Sequence	Experimental Seed Sequence	In Silico Seed Sequence	Beginning of the Seed, In Silico
**NRF2: NC_000002 REGION: complement (177230303..177265131)**					
507	Xq27.3	UUUUGCACCUUUUGGAGUGAA	not specified	TGCAAAA	34449 and 34686 (two binding sites)
634	17q24.2	AACCAGCACCCCAACUUUGGAC	not specified	GCTGGTA	34791
450a-5p	Xq26.3	UUUUGCGAUGUGUUCCUAAUAU	not specified	No binding according to in silico analysis	N/A
129–5p	7q32.1	CUUUUUGCGGUCUGGGCUUGC	not specified	GCAAAAAA	34730 and 34769 (two binding sites)
144–3p	17q11.2	UACAGUAUAGAUGAUGUACU	AUACUGUA	ATACTGTA	34613 and 34718 (two binding sites)
153–3p	2q35	UUGCAUAGUCACAAAAGUGAUC	CUAUGCAA	CTATGCAA	34446
27a-3p	19p13.12	UUCACAGUGGCUAAGUUCCGC	ACUGUGA	ACTGTGA	34410
142–5p	17q22	CAUAAAGUAGAAAGCACUACU	ACUUUAUA	ACTTTATA	34431
28–5p	3q28	AAGGAGCUCACAGUCUAUUGAG	AGCUCCUA	AGCTCCTA	34403
**KEAP1: NC_000019 REGION: complement (10486125..10503356)**					
200a-3p	1p36.33	UAACACUGUCUGGUAACGAUGU	CAGUGUUA	CAGTGTTA	16838
141–3p	12p13.31	UAACACUGUCUGGUAAAGAUGG	CAGUGUUA	CAGTGTTA	16838
7–5p	9q21.32	UGGAAGACUAGUGAUUUUGUUGUU	UGGAAGA	No binding according to in silico analysis	N/A
432–5p	14q32.2	UCUUGGAGUAGGUCAUUGGGUGG	UGGAUGG (exon 2)	TCCAAGA (3′UTR)	16897

**Table 3:** The role of NRF2 in different cancer stem cell models.

Cancer Cell Type	NRF2 Role	Reference
Breast cancer	Regulation of ALDH and contribution to radioresistance	[133]
Ovarian cancer	Regulation of CSC markers, chemoresistance, colony/sphere formation, and tumor growth	[134]
Hepatocellular carcinoma	Promotion of cancer stemness, migration, and expression of ABC transporter genes in sorafenib-resistant cells	[135]
Glioma	Induction of stem markers	[132]
Breast cancer	Antioxidant response	[137]
Breast cancer	Antioxidant response	[138]
Colorectal cancer	Proliferation of differentiated spheroids	[139]
Breast cancer	Self-renewal of breast cancer stem-like cells	[140]
Hepatocellular carcinoma	CSCs enrichment	[141]
Breast cancer	Drug resistance acquisition	[152]
Head and neck cancer	Chemoresistance	[154]
Head and neck cancer	Multidrug resistance	[157]
Ovarian cancer	Drug resistance	[158]
Breast cancer	Resistance to radiation therapy	[160]
Glioblastoma	Resistance to radiation therapy	[161]

## Data Availability

Not applicable.
